# Expression of plasma membrane calcium ATPases confers Ca^2+^/H^+^ exchange in rodent synaptic vesicles

**DOI:** 10.1038/s41598-019-40557-y

**Published:** 2019-03-12

**Authors:** Yoshiyasu Ono, Yasunori Mori, Yoshihiro Egashira, Kenta Sumiyama, Shigeo Takamori

**Affiliations:** 1 Laboratory of Neural Membrane Biology, Graduate School of Brain Science, Doshisha University, 1-3 Tatara-Miyakodani, Kyotanabe, Kyoto 610-0394 Japan; 2 Laboratory for Mouse Genetic Engineering, RIKEN Center for Biosystems Dynamics Research, 1-3 Yamadaoka, Suita, Osaka 565-0871 Japan; 3 Present Address: Department of Physiology, Faculty of Medicine, Osaka Medical College, 2-7 Daigaku-machi, Takatsuki, Osaka 569-8686 Japan

## Abstract

Ca^2+^ transport into synaptic vesicles (SVs) at the presynaptic terminals has been proposed to be an important process for regulating presynaptic [Ca^2+^] during stimulation as well as at rest. However, the molecular identity of the transport system remains elusive. Previous studies have demonstrated that isolated SVs exhibit two distinct Ca^2+^ transport systems depending on extra-vesicular (cytosolic) pH; one is mediated by a high affinity Ca^2+^ transporter which is active at neutral pH and the other is mediated by a low affinity Ca^2+^/H^+^ antiporter which is maximally active at alkaline pH of 8.5. In addition, synaptic vesicle glycoprotein 2 s (SV2s), a major SV component, have been proposed to contribute to Ca^2+^ clearance from the presynaptic cytoplasm. Here, we show that at physiological pH, the plasma membrane Ca^2+^ ATPases (PMCAs) are responsible for both the Ca^2+^/H^+^ exchange activity and Ca^2+^ uptake into SVs. The Ca^2+^/H^+^ exchange activity monitored by acidification assay exhibited high affinity for Ca^2+^ (*K*_m_ ~ 400 nM) and characteristic divalent cation selectivity for the PMCAs. Both activities were remarkably reduced by PMCA blockers, but not by a blocker of the ATPase that transfers Ca^2+^ from the cytosol to the lumen of sarcoplasmic endoplasmic reticulum (SERCA) at physiological pH. Furthermore, we rule out the contribution of SV2s, putative Ca^2+^ transporters on SVs, since both Ca^2+^/H^+^ exchange activity and Ca^2+^ transport were unaffected in isolated vesicles derived from SV2-deficient brains. Finally, using a PMCA1-pHluorin construct that enabled us to monitor cellular distribution and recycling properties in living neurons, we demonstrated that PMCA1-pHluorin localized to intracellular acidic compartments and recycled at presynaptic terminals in an activity-dependent manner. Collectively, our results imply that vesicular PMCAs may play pivotal roles in both presynaptic Ca^2+^ homeostasis and the modulation of H^+^ gradient in SVs.

## Introduction

Synaptic vesicles (SV) are storage organelles for neurotransmitters and play a key role in synaptic transmission. Vesicular transport of classical neurotransmitters including glutamate, GABA/glycine, biogenic monoamines, acetylcholine, and adenosine triphosphate (ATP) is mediated by the respective vesicular transporters for these neurotransmitters. Vesicular transport is driven by a proton electrochemical gradient generated by the vacuolar-type H^+^ ATPase (V-ATPase)^1^. The V-ATPase generates both a pH gradient (ΔpH) and a membrane potential (internal positive voltage, Δ**Ψ**) across the SV membrane. Depending on the chemical properties of neurotransmitters and the intrinsic characteristics of vesicular transporters, the dependence of neurotransmitter uptake on either driving force varies among the neurotransmitters. In principle, the uptake of cationic transmitters such as monoamines and acetylcholine depends primarily on ΔpH, whereas the uptake of anionic transmitters such as glutamate and ATP depends more on Δ**Ψ**, although pivotal roles of protons in glutamate transport have also been suggested^2–4^. The uptake of zwitter-ionic transmitters such as GABA and glycine depends equally on both ΔpH and Δ**Ψ** (but see Juge *et al*.^5^ who proposed a predominant role of Δ**Ψ**). Therefore, a balance between ΔpH and Δ**Ψ** across individual SVs differentially regulate transport rates and storage of neurotransmitters in SVs^1^.

The balance between ΔpH and Δ**Ψ** is regulated by the permeability of the SV membrane to various charged ions and their concentration gradients^6^. In fact, SVs contain various transporters and/or channels for ions that directly or indirectly modulate H^+^ flux across SVs. For instance, it has been demonstrated that SVs exhibit a Cl^−^ conductance that serves a shunting current for H^+^, resulting in an increase in net proton influx and thus a generation of larger ΔpH^7,8^. In the case of SVs that store glutamate, the vesicular glutamate transporters (VGLUTs) are proposed to confer the Cl^−^ flux^2,3,9,10^ (but see also Juge *et al*.^11^). The contribution of the chloride channel (CLC) family has been proposed, especially for SVs that transport other neurotransmitters, but their physiological relevance remains controversial^12–15^. More recently, cation/H^+^ exchange activity conferred by either Na^+^/H^+^ exchangers (NHEs)^16^ or VGLUTs^10^ (in the case of glutamate-containing vesicles) were shown to decrease ΔpH and consequently facilitate Δ**Ψ**. In stark contrast, the contribution of divalent cations such as Ca^2+^ and Zn^2+^ has received less attention. To date, the mechanism and molecular entity of the transporting molecule(s) remain elusive.

Classical studies using SV fraction isolated from the electric organ of *Torpedo marmorata* revealed that SVs exhibited an ATP-dependent active Ca^2+^ transport activity^17–19^. Consistent with this, a transient increase of Ca^2+^ in the SV lumen was observed after stimulation at the cholinergic synapses of the electric organ of *Torpedo marmorata*^20^, suggesting that SVs can function as a Ca^2+^ store at presynaptic terminals in a physiological context. Efforts to decipher the Ca^2+^ transport protein(s) in mammalian SVs have revealed that at least two distinct mechanisms may exist: vanadate-sensitive P-type Calcium ATPases, which are fully active at neutral pH, and a molecularly unidentified Ca^2+^/H^+^ antiporter, which functions maximally at alkaline pH of 8.5^21–23^. These two transport systems exhibit substantial differences in their affinities for Ca^2+^. While *K*_m_ of the vanadate-sensitive Ca^2+^ ATPases lies in the order of several hundred nM, *K*_m_ of the Ca^2+^/H^+^ antiporter exceeds 300 µM, which far surpasses physiological Ca^2+^ concentrations at presynaptic terminals^24^. Moreover, these two transport systems exhibit characteristic dependence on Ca^2+^ concentrations. Notably, Ca^2+^ ATPase activity increases with Ca^2+^ concentration in the medium, reaching a maximum at ~25 µM, whereas its activity is inhibited at higher Ca^2+^ concentrations and is completely abolished at ~500 µM Ca^2+^ ^22^.

Aside from these two SV Ca^2+^ transport systems, it has long been conjectured that one of the major SV proteins, SV2s (synaptic vesicle glycoprotein 2s), may function as a Ca^2+^ transporter in SVs^25^, because the genetic deletion of SV2s revealed synaptic phenotypes that could be well-explained by the increase in cytoplasmic [Ca^2+^]^26,27^ (but see^28^). Nevertheless, the relative contributions of these proteins to Ca^2+^ transport into SVs has not been elucidated, and whether/how the activities of these proteins may affect ΔµH^+^ to influence neurotransmitter uptake remains unclear.

In this study, we aimed to clarify the molecular identity of the Ca^2+^ transporting protein(s) in SVs and how their activity influences ΔµH^+^ of SVs. Using a fast Ca^2+^ chelator BAPTA, instead of EGTA that was previously used to control free Ca^2+^ concentrations in the buffer^21,29^, we show that SVs isolated from rodent brains exhibit high affinity Ca^2+^/H^+^ exchange activity even at neutral pH that would attenuate ΔpH in SVs. Biochemical properties of the exchanger such as the affinity for Ca^2+^, ion selectivity, and sensitivity to specific inhibitors resemble those of the plasma membrane Ca^2+^ ATPases (PMCAs). Furthermore, we ruled out a contribution of SV2s to Ca^2+^ uptake into SVs by analyzing vesicle fractions from SV2-deficient brains. We further demonstrated using a pH-sensitive fluorescent protein (pHluorin)^30^ as a reporter that PMCA1, an isoform of PMCA expressed in the mammalian nervous system, indeed resides mainly on SVs and is capable of recycling at presynaptic terminals in an activity-dependent manner. Given the predominant role of PMCAs in regulating Ca^2+^ homeostasis at presynaptic terminals^31^, our results imply that vesicular PMCAs play dual roles at presynaptic terminals in Ca^2+^ homeostasis and in modulating ΔµH^+^ in SVs via Ca^2+^/H^+^ antiport activity.

## Results

### A Ca^2+^/H^+^ exchanger in SVs operates at neutral pH

**Figure 1:**
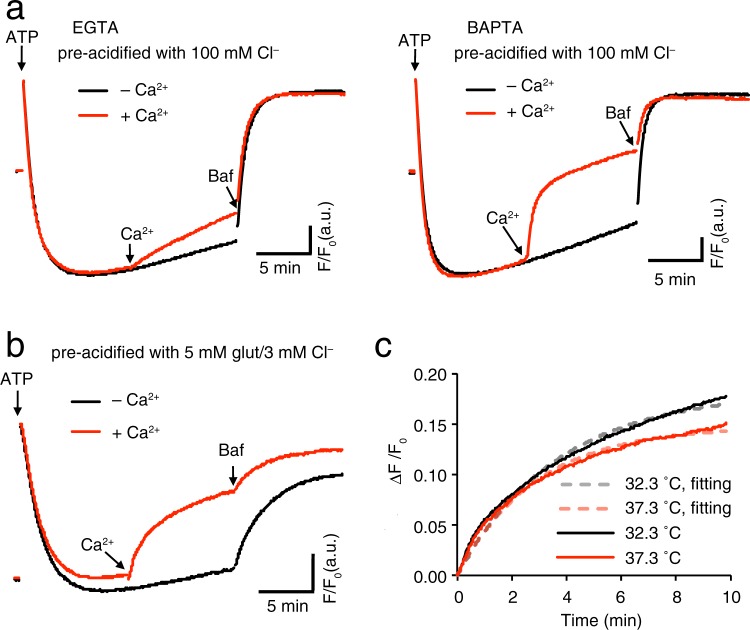
Ca^2+^/H^+^ exchange activity attenuates ΔpH in synaptic vesicles at neutral pH. (**a**) Effect of Ca^2+^ in the presence of EGTA (left) or BAPTA (right) on ATP-dependent acidification at pH 7.2. Acidification was measured using acridine orange as a reporter dye. Crude SV fraction (LP2) was incubated in the presence of 50 µM EGTA or 50 µM BAPTA in the presence of 100 mM KCl. To initiate acidification, 2 mM ATP was added, and 600 µM CaCl_2_ (only for red traces) and 500 nM Bafilomycin (Baf) were added at time points indicated by arrows. Note that de-quenching (indicated by increases in fluorescence) upon the addition of CaCl_2_ was greater in the presence of BAPTA than EGTA. The traces were the representative traces from more than three measurements. (**b**) Effect of Ca^2+^ in the presence of BAPTA on ATP-dependent, glutamate-induced acidification at pH 7.2. Crude SV fraction (LP2) was incubated in the presence of 50 µM BAPTA in the presence of 5 mM glutamate and 3 mM KCl. To initiate acidification, 2 mM ATP was added, and 600 µM CaCl_2_ (only for red traces) and 500 nM bafilomycin (Baf) were added at time points indicated by arrows. The traces were the representative traces from more than three measurements. (**c**) Temperature coefficient (Q_10_) of Ca^2+^-induced alkalization. Ca^2+^-induced alkalization of SVs that were pre-acidified in the presence of 100 mM KCl was monitored either at 32.3 °C (black) or at 37.3 °C (red). The alkalization kinetics were obtained by exponential fittings shown in dotted lines. Q_10_ was calculated as described in Materials and Methods.

Evidence that SVs isolated from sheep brain cortex contain low affinity Ca^2+^/H^+^ antiport activity relied essentially on an acidification assay in which fluorescence quenching of acridine orange due to V-ATPase-dependent acidification was reversed by addition of Ca^2+^ ^21–23^. This activity was characterized by its low affinity (*K*m > 200 µM) and operated at pH 8.5. However, chelation of Ca^2+^ by EGTA would result in rapid liberation of H^+^ from EGTA, and the ability of Ca^2+^ chelation by EGTA depends critically on pH. As such, addition of Ca^2+^ in the presence of EGTA during the measurement may have caused a change in pH, raising the possibility that the affinity and pH dependence may have been disturbed. Therefore, we decided to use BAPTA whose *K*_D_ is similar to that of EGTA, but the ability to chelate Ca^2+^ is much less affected by pH changes. First, we tested the effect of Ca^2+^ in the presence of BAPTA at neutral pH of 7.2, and compared with that in the presence of EGTA (Fig. 1a). Acidification of crude SV fraction (LP2) was induced by adding 2 mM ATP in the presence of 100 mM Cl^−^, and 600 µM Ca^2+^ was then added. As observed in previous studies at pH 8.5^21,23^, slow alkalization of SVs was also observed in the presence of EGTA (Fig. 1a, red trace in the left panel). Notably, the effect of Ca^2+^ was more pronounced when the assay was performed in the presence of BAPTA (Fig. 1a, red trace in the right panel), indicating that the Ca^2+^/H^+^ exchange activity was also operative at neutral pH. Essentially, the same results were obtained under conditions where acidification was monitored in SVs pre-acidified by VGLUT-mediated glutamate transport^3^ (Fig. 1b), confirming that the activity originated from SVs rather than contaminating organelles such as microsomes and mitochondria. To further confirm if the de-quenching represented a transporter-mediated process, we measured de-quenching velocities upon the addition of Ca^2+^ at various temperatures (32.3 °C and 37.3 °C) under the same conditions as those in Fig. 1b (right panel). The temperature co-efficient (Q_10_) was estimated to be ~1.6 (Fig. 1c), which is within the range of physical properties of transporter-mediated processes rather than simple diffusion^32,33^.

### The vesicular Ca^2+^/H^+^ exchanger exhibits high affinity for Ca^2+^

**Figure 2:**
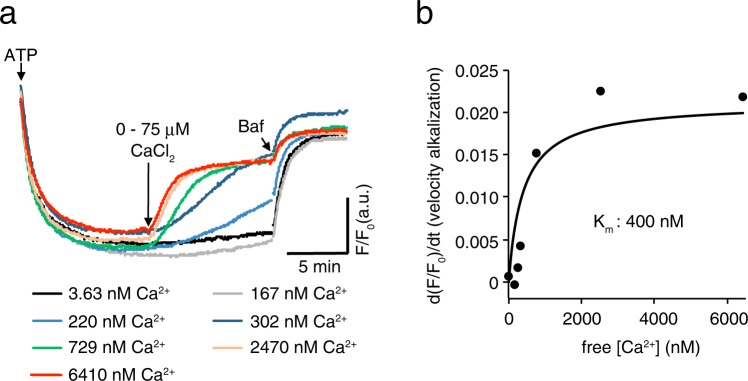
Apparent affinity of the Ca^2+^/H^+^ exchanger on SVs. (**a**) Alkalization of SVs by various concentrations of Ca^2+^. After SVs were maximally acidified in the presence of 100 mM KCl, various concentrations of CaCl_2_ were added. Individual traces in the presence of various free [Ca^2+^], ranging from 3.63–6410 nM of free [Ca^2+^] were color-coded. (**b**) The initial slopes of alkalization in (**a**) were plotted against free [Ca^2+^]. Data points were fitted with the Michaelis-Menten equation to calculate *K*_m_.

**Figure 3:**
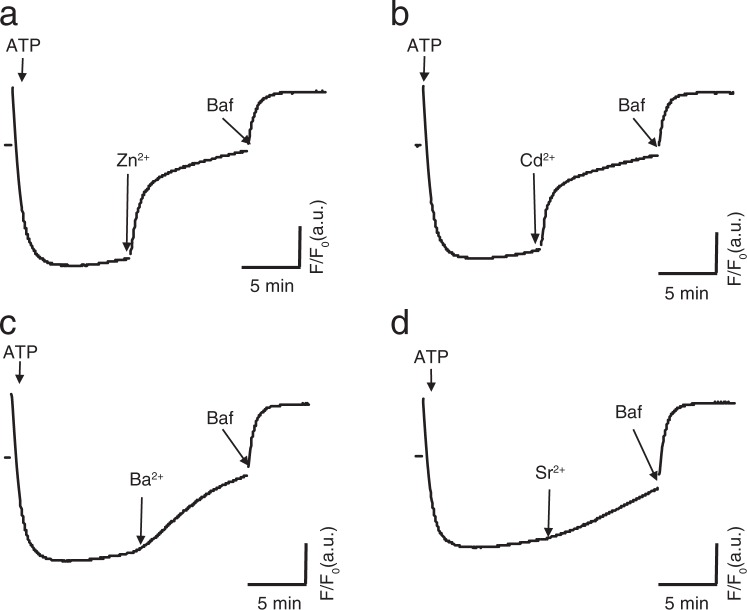
Selectivity of the Ca^2+^/H^+^ exchanger for other divalent cations. LP2 fraction was acidified in the presence of 100 mM KCl at pH 7.2. After stable baselines were achieved, various divalent cations including Zn^2+^, Cd^2+^, Ba^2+^, and Sr^2+^ at final concentration of 50 µM were added. The traces were the representative traces from more than three measurements. Note that we observed the same results when vesicles were pre-acidified in the presence of 5 mM glutamate and 3 mM KCl (see Supplementary Fig. 2).

To estimate the affinity of the Ca^2+^/H^+^ exchanger in SVs at neutral pH, we measured alkalization of pre-acidified SVs by various concentrations of CaCl_2_ in the presence of a fixed concentration of BAPTA (50 µM). To this end, we first estimated free Ca^2+^ concentrations in our assay buffers using fura-2 as a Ca^2+^ indicator and found it to be ~1 µM (Supplementary Fig. 1). Taking this into account, free Ca^2+^ concentrations in the assay buffer were set by adding CaCl_2_ (see Materials and Methods for details). After complete acidification of SVs in the presence of 100 mM KCl was achieved, we added a series of Ca^2+^ concentrations ranging from 0 to 75 µM that were equivalent to 3.63 to 6410 nM free [Ca^2+^] in the assay medium. With the ranges of Ca^2+^ added, the rate of alkalization increased as a function of free Ca^2+^ concentrations (Fig. 2a). A plot of initial velocities of alkalization as a function of free Ca^2+^ concentrations revealed that *K*_m_ of Ca^2+^-induced SV alkalization was ~400 nM (Fig. 2b), a value that appeared to be several magnitudes lower than that reported previously^21^. To further characterize the Ca^2+^/H^+^ antiport activity at neutral pH, we tested a series of divalent cations for the ability to alkalize pre-acidified SVs. Previous studies demonstrated that, at alkaline pH, Zn^2+^ and Cd^2+^ are substrates of the same Ca^2+^/H^+^ exchanger, whilst Ba^2+^ and Sr^2+^ are not^29^. In our assay conditions in the presence of BAPTA at pH 7.2, Zn^2+^and Cd^2+^ induced alkalization of pre-acidified SVs in the presence of 100 mM KCl to a similar extent as that of Ca^2+^, whereas Ba^2+^ induced slower but significant alkalization (Fig. 3). Consistent with previous results, Sr^2+^ showed marginal alkalization, indicating that the Ca^2+^/H^+^ exchanger in SVs shows substrate preference as follows: Ca^2+^ ≈ Zn^2+^ ≈ Cd^2+^ > Ba^2+^ ≫ Sr^2+^. The same trend was observed when LP2 was pre-acidified by glutamate, indicating that the alkalization by divalent cations originated from VGLUT-containing SVs (Supplementary Fig. 2). It should be noted here that it remains unknown if these divalent cations are transported by distinct transporters with similar proton coupling, or if the alkalization resulted from the blockade of the V-ATPase activity (see also Supplementary Fig. 4 related to Fig. 5).

### SV2s are not responsible for Ca^2+^/H^+^ exchange in SVs

As described in the Introduction, there are several candidates responsible for Ca^2+^ transport in SVs. Comprehensive proteomic analysis of purified SV fraction identified two P-type ATPases, PMCA and and one that exchanges Ca^2+^ from the cytosol with protons in th elumen of sarcoplasmic endoplasmic reticulum (SERCA)^34^. However, contributions of these ATPases to SV acidity due to a H^+^ coupling have not been directly investigated. Furthermore, according to synaptic phenotypes related to changes in presynaptic Ca^2+^ concentrations and in presynaptic calcium buffering in SV2-deficient neurons^26,27^, it has been hypothesized that SV2s may be involved in Ca^2+^ transport into SVs. We therefore sought to clarify which protein was responsible for the observed Ca^2+^/H^+^ exchange activity in SVs at neutral pH.

**Figure 4:**
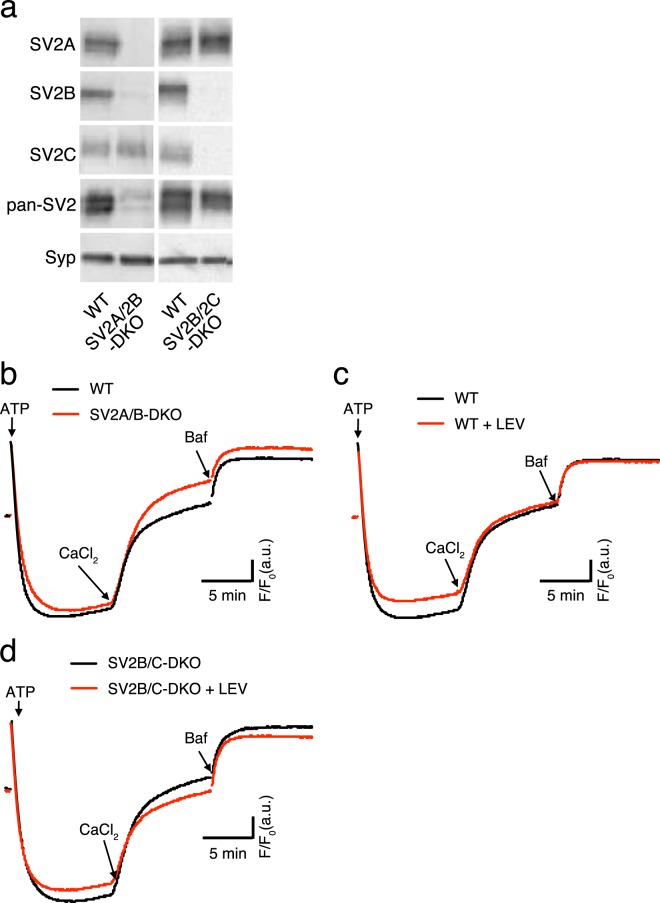
SV2s do not confer Ca^2+^/H^+^ exchange. (**a**) Expression of SV2 isoforms in synaptic vesicles derived from SV2A/B-DKO and SV2B/C-DKO mice. Equal amounts of vesicle proteins from each genotype (20 µg/lane) were analyzed by western blotting using isoform-specific antibodies. Antibodies used for western blots are indicated at the left of the images. Rabbit polyclonal antibodies against SV2A, SV2B, and SV2C, and a mouse monoclonal antibody that recognizes all SV2 isoforms (pan-SV2) were used. For loading controls, a mouse monoclonal antibody against synaptophysin (Syp) (Cl7.2) was used. Note that expression of the SV2 isoforms was completely abolished in the respective DKO samples. A faint band revealed by the pan-SV2 antibody in the SV2A/2B-DKO sample indicated that SV2C content was much less than that of SV2A or SV2B. The images were cropped from four independent blots for presentation, and the original digital images of the full-length blots are presented in Supplementary Fig. 3. (**b**) Ca^2+^-induced alkalization in vesicles derived from SV2A/B-DKO (red) compared to wild-type mice (black). 50 µM CaCl_2_ was added after vesicles were pre-acidified in the presence of 100 mM KCl. (**c**) Effect of levetiracetam (LEV, 30 µM) on Ca^2+^-induced alkalization in vesicles from wild-type mice. Vesicles were pre-acidified in the presence of 100 mM KCl, and 50 µM CaCl_2_ was then added. LEV pre-treatment (red traces) shows little impact on alkalization by Ca^2+^ compared to the respective controls without LEV pre-treatment (black traces). (**d**) Effect of levetiracetam (LEV, 30 µM) on Ca^2+^-induced alkalization in vesicles from SV2B/2C-DKO mice. Measurements were performed as in (**c**). LEV pre-treatment (red traces) shows little impact on alkalization by Ca^2+^ compared to the respective controls without LEV pre-treatment (black traces). Traces in (**b**–**d**) are the representative data from two to three measurements in each condition.

First, in order to verify the contribution of SV2s in Ca^2+^/H^+^ exchange, we generated SV2A/2B- and SV2B/2C-double knockout (DKO) mice using the CRISPR/Cas9 system. We then tested if Ca^2+^/H^+^ exchange activity was affected. Western blot analysis of LP2 fractions purified from SV2A/2B-DKO or SV2B/2C-DKO mice using isoform-specific antibodies confirmed efficient gene knockout by the CRISPR/Cas9 system (Fig. 4a). Moreover, western blot analysis using a pan-SV2 antibody that equally recognizes all SV2 isoforms revealed that SV2A and SV2B represented the major SV2 isoforms in brain, and expression of SV2C was minor (Fig. 4a). As a control, we confirmed that expression of synaptophysin, an abundant SV protein^34^, was unaltered in SVs derived from SV2-deficient mice. As shown in Fig. 4b, Ca^2+^/H^+^ exchange activity was normal in SVs from SV2A/2B-DKO mice, and the extent of alkalization induced by 50 µM CaCl_2_ was comparable to that observed in SVs from wild-type mice of the same age (Fig. 4b).

Levetiracetam (LEV, also known as Keppra® or E Keppra®) is an established second-generation antiepileptic drug, whose molecular target is SV2A^35^. LEV selectively binds to the cytoplasmic portion of SV2A^35,36^ and potentially inhibits SV2A function^36^. The inability of LEV to inhibit Ca^2+^/H^+^ exchange (Fig. 4c) in SVs from wild-type mice provided further support that SV2A was not responsible for the activity, although SV2B, another isoform of SV2s, was present in the SV preparation. To completely rule out the contribution of SV2A to Ca^2+^/H^+^ exchange activity, we examined the effect of LEV on transport activity in SV2B/2C-DKO samples (note that only SV2A is present in SV2B/2C-DKO mice). Again, LEV did not reduce Ca^2+^-induced alkalization (Fig. 4d). Taken together, although SV2s have been proposed to function as Ca^2+^ transporters in SVs, SV2s do not contribute to high affinity Ca^2+^/H^+^ exchange activity at neutral pH.

### The plasma membrane Ca^2+^ ATPases (PMCAs) mediate Ca^2+^/H^+^ exchange in SVs

**Figure 5:**
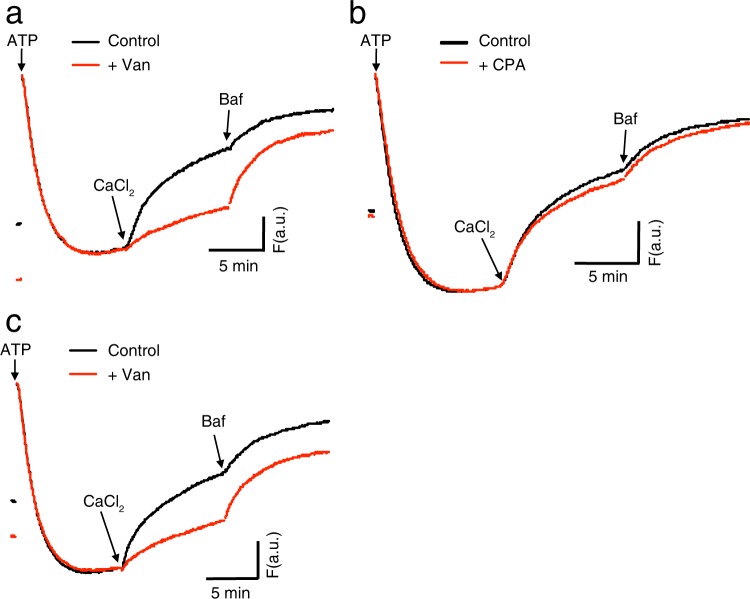
PMCAs predominantly contribute to Ca^2+^/H^+^ exchange. (**a**) Effect of vanadate (Van, 500 µM) on Ca^2+^-induced alkalization. Vesicles were pre-acidified in the presence of 5 mM glutamate and 3 mM KCl in the absence (black) and presence (red) of 500 µM Van, and 50 µM CaCl_2_ was added to induce alkalization. (**b**) Effect of CPA (CPA, 15 µM) on Ca^2+^-induced alkalization. Vesicles were pre-acidified in the presence of 5 mM glutamate and 3 mM KCl in the absence (black) and presence (red) of 15 µM CPA, and 50 µM CaCl_2_ was added to induce alkalization. (**c**) Effect of vanadate (Van, 500 μM) on Ca^2+^-induced alkalization by 500 µM CaCl_2_. Measurements were performed as in (**a**) except that a high concentration of CaCl_2_ (500 µM) was added. All traces are the representative data from at least two to three measurements in each condition. Since vanadate slightly reduced AO quenching signals in the presence of 5 mM glutamate and 3 mM KCl, fluorescence traces were normalized by amplitudes of acidification, and the starting points of alkalization by CaCl_2_ were adjusted for comparison.

Next, we examined the effect of blockers that selectively inhibit the P-type Ca^2+^ ATPases, including PMCAs and SERCA. Among the available inhibitors, 500 µM vanadate, a PMCA blocker, potently inhibited Ca^2+^-induced alkalization of SVs (Fig. 5a). On the other hand, 15 µM cyclopiazonic acid (CPA), a SERCA inhibitor^37^, did not affect alkalization by Ca^2+^ (Fig. 5b), indicating that the high affinity Ca^2+^/H^+^ exchange activity observed with this assay was attributed to PMCAs. Notably, whereas Ba^2+^- and Sr^2+^-induced alkalization of pre-acidified SVs by glutamate was largely abolished in the presence of vanadate, alkalization induced by Zn^2+^ or Cd^2+^ was not completely blocked by vanadate (Supplementary Fig. 4). These observations implied that, while Ba^2+^ and Sr^2+^ are recognized by PMCAs, there are other divalent cation/H^+^ exchangers that transfer Zn^2+^ or Cd^2+^. Of note, ZnT-3, a vesicular Zn^2+^ transporter, is present on a subpopulation of SVs that contain glutamate^38–40^, and therefore could contribute to vanadate-insensitive Zn^2+^/H^+^ exchange activity observed here, although the precise mechanism of Zn^2+^ transport into SVs, e.g. proton coupling during transport cycles, remains to be determined.

It was previously reported that in sheep SVs, vanadate-sensitive Ca^2+^ transport activity was largely absent in the presence of >200 µM Ca^2+^ ^22^. However, Ca^2+^-induced alkalization at neutral pH was evident by 500 µM Ca^2+^ under our assay conditions (Fig. 5c, black trace). More importantly, 500 µM vanadate effectively impeded the alkalization (Fig. 5c, red trace), indicating that even in the presence of higher concentrations of Ca^2+^, Ca^2+^-induced alkalization was mediated by PMCAs, irrespective of the Ca^2+^ concentrations within this range.

### The PMCAs, but not SV2s, are responsible for the majority of Ca^2+^ transport into SVs at neutral pH

**Figure 6:**
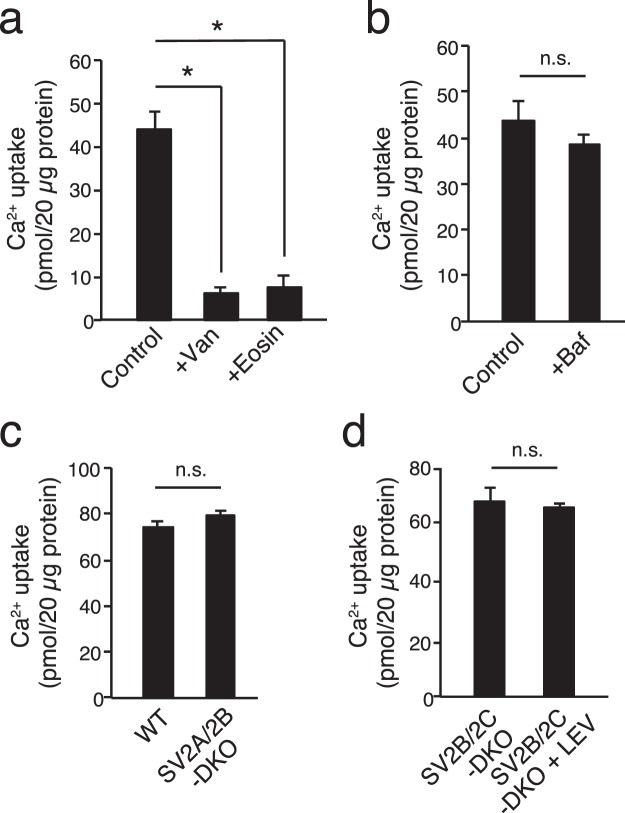
PMCAs predominantly contribute to Ca^2+^ uptake into SVs independent of H^+^ electrochemical gradient generated by V-ATPase. (**a**) Ca^2+^ transport was measured using ^45^Ca^2+^ as a tracer. The assay was performed in the absence of inhibitors (Control) or in the presence of vanadate (Van, 500 µM) or eosin (10 µM). Both PMCA blockers effectively inhibited Ca^2+^ transport into the SV fraction. Error bars indicate s.e.m. of three independent measurements. *p < 0.0025, unpaired equal variance Student’s *t*-test. (**b**) Ca^2+^ transport was measured as in (a) in the absence (Control) and presence of the V-ATPase inhibitor, bafilomycin A (500 nM) (Baf). Error bars indicate s.e.m. of three measurements. n.s. indicates not significant (*p* > 0.1, unpaired equal variance Student’s *t*-test). (**c**) Ca^2+^ transport into LP2 from wild-type mice (WT) and that from SV2A/2B-DKO mice were measured. Error bars indicate s.e.m. of three measurements. n.s. indicates not significant (*p* > 0.1, unpaired equal variance Student’s *t*-test). (**d**) Ca^2+^ transport into LP2 from SV2B/2C-DKO mice in the absence or presence of 30 µM levetiracetam (LEV) were compared. Error bars indicate s.e.m. of three measurements. n.s. indicates not significant (*p* > 0.1, unpaired equal variance Student’s *t*-test).

To verify if Ca^2+^-induced alkalization observed in our assay conditions was correlated with Ca^2+^ transport by PMCAs, we next examined radioactive ^45^Ca^2+^ uptake in the presence of various inhibitors. For this purpose, LP2 fraction was further purified by applying it to sucrose gradient centrifugation to minimize the contamination of other organelles and membranes. Consistent with the previous report^22^, ^45^Ca^2+^ uptake was suppressed by PMCA inhibitors (10 µM eosin and 500 µM vanadate) to background levels measured in the absence of ATP (Fig. 6a). In contrast, the V-ATPase inhibitor, bafilomycin (500 nM), did not affect ^45^Ca^2+^ uptake (Fig. 6b). Collectively, these results indicate that PMCAs are responsible for the majority of Ca^2+^ transport into SVs. In agreement with the results from acidification assays using SV2-KO vesicles, ^45^Ca^2+^ uptake into SVs derived from SV2A/SV2B-DKO mice (which lack the majority of SV2 proteins) was not significantly different from that derived from wild-type mice (Fig. 6c). Likewise, addition of 30 µM LEV did not reduce Ca^2+^ transport into SVs derived from SV2B/2C-DKO mice (Fig. 6d), excluding the possibility that SV2s play a role in Ca^2+^ transport into SVs.

### PMCA1-pHluorin localizes in acidic compartments at presynaptic terminals

**Figure 7:**
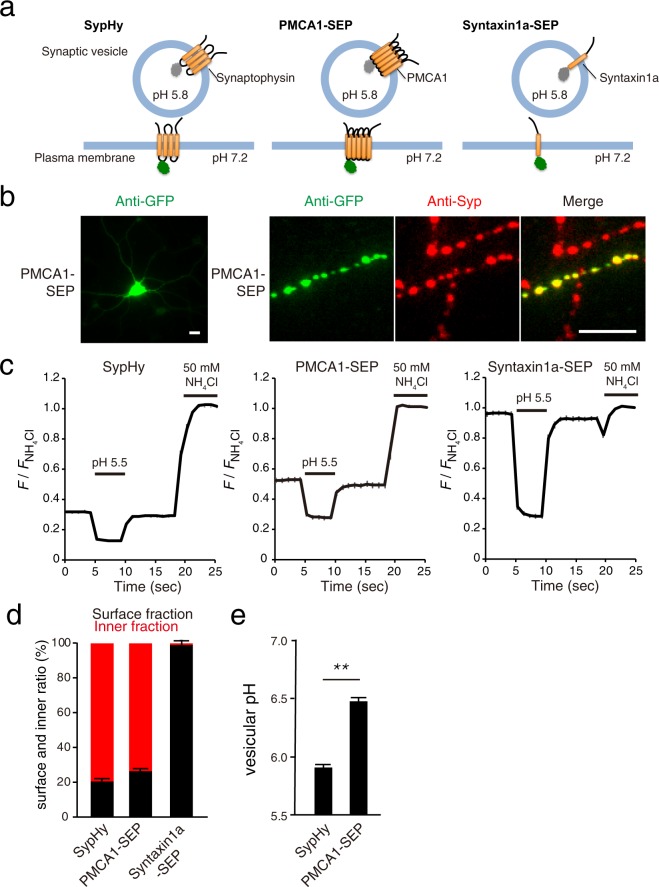
Localization of PMCA1-pHluorin in acidic compartments at presynaptic terminals of cultured hippocampal neurons. (**a**) Schematic diagrams of the pHluorin probes. The super-ecliptic pHluorin (SEP) was fused either to the second luminal loop of synaptophysin (SypHy), to the first luminal loop of PMCA1 (PMCA1-SEP), or to the C-terminal end of syntaxin1a (Syntaxin1a-SEP). When these proteins reside on synaptic vesicle membranes, the SEP fluorescence is quenched due to the acidic pH (~5.8), whereas they become fluorescent when they are present at the plasma membrane and are thereby exposed to the extracellular neutral pH (~7.2). (**b**) Fluorescence images of cultured hippocampal neurons expressing PMCA1-SEP. PMCA1-SEP was visualized by immunostaining with rabbit polyclonal anti-GFP antibody (green). A synaptic vesicle marker, synaptophysin, was visualized by immunostaining with mouse monoclonal anti-synaptophysin antibody (red). The right panels show merged pictures of the two immunostainings. Scale bars indicate 10 µm (cell body in the left panel) and 5 µm (right panels). (**c**) Average fluorescence of SypHy (left, n = 100 boutons from 10 images), PMCA1-SEP (middle, n = 99 from 10 images), and Syntaxin1a-SEP (right, n = 100 from 10 images) in response to sequential application of a pH 5.5 solution and 50 mM NH_4_Cl. After baseline subtraction, fluorescence of bouton-like structures were normalized to those during 50 mM NH_4_Cl application. (**d**) Distribution of SypHy, PMCA1-SEP, and Syntaxin1a-SEP between cell surface (black bars) and acidic intracellular compartment (red bars) deduced from the traces in (**c**). Error bars indicate s.e.m. for the cell surface fraction. (**e**) Luminal pHs of vesicles carrying SypHy or PMCA1-SEP deduced from the traces in (**c**). Error bars indicate s.e.m. **p < 0.00025, unpaired unequal variance Student’s *t*-test.

The biochemical data described have demonstrated that PMCAs were responsible for the majority of Ca^2+^ transport into SV-rich membrane fractions, but it remains unclear whether they localized in functionally competent SVs in living neurons. In fact, isolated SVs from rodent brains contain various endosomal proteins such as endosomal SNAREs and rab proteins that may preferentially localize non-recycling vesicles at presynaptic terminals^34^. To clarify whether PMCAs localized at recycling or non-recycling vesicles in living neurons, we constructed a fluorescent reporter in which a pH-sensitive green fluorescent protein (super-ecliptic pHluorin; SEP^30^) was conjugated to the first luminal loop of PMCA1 (Fig. 7a). A SEP fused to the second luminal loop of synaptophysin (SypHy) and a SEP fused to the C-terminal end of Syntaxin1a (Syntaxin1a-SEP) were used as controls for SV residents and plasma membrane residents, respectively (Fig. 7a). Due to the pH sensitivity of the reporter, fluorescence is quenched in acidic compartments whereas it rises when the reporter is exposed to the neutral pH solution in the extracellular space^30^. When PMCA1-SEP was transiently expressed in cultured hippocampal neurons derived from embryonic mouse brains, immunostaining of the fixed cells with an anti-GFP antibody revealed ubiquitous expression of PMCA1-SEP in cell bodies and dendrites (Fig. 7b, left), as well as in presynaptic bouton-like structures (Fig. 7b, right). Co-immunostaining with an antibody against synaptophysin further confirmed the presynaptic localization of PMCA1-SEP (Fig. 7b, right panels). Estimation of inner/surface distribution of PMCA1-SEP by sequential applications of an acidic solution (pH 5.5) and a 50 mM NH_4_Cl solution^41^ indicated that ~75% of PMCA1-SEP was localized in the acidic compartments whose average pH was 6.47 ± 0.03 (Fig. 7d,e, Supplementary Fig. 5). Control experiments with SypHy revealed that ~80% of SypHy was localized in the acidic compartments whose average luminal pH was ~5.91 ± 0.02. In contrast, control experiments with Syntaxin1a-SEP revealed that it was localized almost exclusively on the plasma membrane (99.5 ± 2.2%) (Fig. 7c–e, Supplementary Fig. 5). The higher luminal pH of PMCA1-pHluorin-positive vesicles indicates that a portion of PMCA1-pHluorin may localize in endosomal compartments. Alternatively, exogenous expression of PMCA1-pHluorin may exert additional Ca^2+^/H^+^ antiport, which would facilitate alkalization of the vesicular lumen. Collectively, although PMCA1-SEP expression was not restricted to the presynaptic terminals in cultured hippocampal neurons, a fraction of PMCA1-SEP did seem to be localized in the acidic compartments whose luminal pH was similar to that of typical SVs.

### PMCA1-SEP recycles at presynaptic terminals

**Figure 8:**
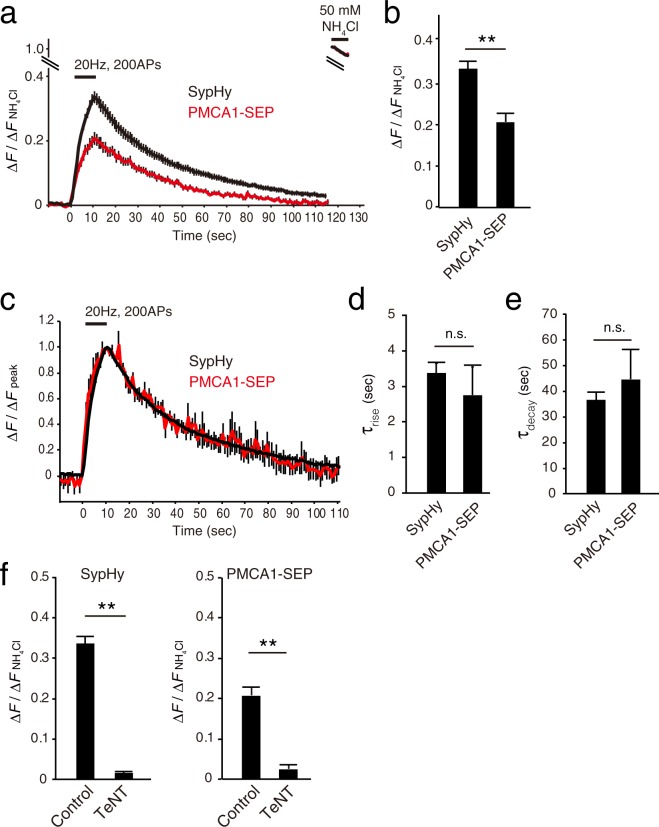
Activity-dependent recycling of PMCA1-pHluorin at presynaptic terminals. (**a**) SypHy (black; n = 100 boutons from 10 images) and PMCA1-SEP (red; n = 53 from 6 images) fluorescence in response to 200 action potentials (APs) at 20 Hz. Fluorescence signals were normalized to those obtained during NH_4_Cl application at the end of recordings. (**b**) Peak fluorescence of SypHy and PMCA1-SEP at the end of field stimulation. Error bars indicate s.e.m. **p < 0.00025, unpaired unequal variance Student’s *t*-test. (**c**) Superimposed traces of SypHy (black) and PMCA1-SEP (red). The traces in (**a**) were normalized to the peak fluorescence at the end of repetitive stimulation. (**d**) Time constants of the rise time during stimulation. Exocytic rise phases were fitted with single exponentials and time constants (τ_rise_) were deduced by using the Solver function in Excel software. An average from one image was taken as n = 1. Error bars indicate s.e.m. n.s indicates *p* > 0.1, unpaired equal variance Student’s *t*-test. (**e**)Time constants of the decay phases after the cessation of stimuli. Decay traces were fitted with single exponentials and endocytic time constants (τ_decay_) were deduced using the Solver function in Excel software. An average from one image was taken as n = 1. Error bars indicate s.e.m. n.s indicates *p* > 0.1, unpaired equal variance Student’s *t*-test. (**f**) SypHy (left; n = 88 boutons from 9 images) and PMCA1-SEP (right; n = 72 boutons from 8 images) fluorescence in response to 200 APs at 20 Hz without (Control) or with 16 hours tetanus toxin treatment (TeNT, 10 nM). Bars indicate peak fluorescence of SypHy and PMCA1 at the end of stimulation with or without tetanus toxin pretreatment. Error bars indicate s.e.m. **p < 0.00025, unpaired unequal variance Student’s *t*-test.

To examine whether PMCA1-SEP-positive vesicles would recycle at presynaptic terminals in an activity-dependent manner, cultured hippocampal neurons expressing PMCA1-SEP were exposed to repetitive electrical stimulation (Fig. 8). At the end of recordings, 50 mM NH_4_Cl solution was applied to estimate the total fluorescence of the SEP molecules (Fig. 8a). Notably, PMCA1-SEP fluorescence increased upon repetitive stimulation at 20 Hz for 10 sec in a manner similar to that of SypHy (Fig. 8a), although the fraction of PMCA1-SEP molecules engaged in exocytosis during stimulation was significantly less than that of SypHy (Fig. 8b). In accordance with the slightly higher vesicular pH of the PMCA1-carrying vesicles (Fig. 7e), this indicated that compared to synaptophysin, more PMCA1 was localized in non-recycling acidic compartments such as endosomes. The kinetics of the rise phase (mainly reflecting exocytosis) and decay phase (reflecting endocytosis and subsequent re-acidification of endocytosed vesicles) were identical to those of SypHy (Fig. 8c-e). Furthermore, when neurons were treated with 10 nM tetanus toxin (TeNT), which cleaves the major vesicular SNARE (v-SNARE) synaptobrevin/VAMP2^42^, PMCA1-SEP responses were completely abolished (Fig. 8f, Supplementary Fig. 6). This effect was also observed for SypHy (Fig. 8f, Supplementary Fig. 6), indicating that PMCA1 was present on synaptobrevin/VAMP2-positive vesicles and followed the same fate as that of synaptobrevin/VAMP2 during activity-dependent SV recycling.

## Discussion

In this study, we characterized the biochemical properties of Ca^2+^ transport into SVs isolated from rodent brains. We found that PMCAs, the plasma membrane Ca^2+^ ATPases, confer the majority of Ca^2+^ transport into SVs. Previous studies have indicated that there are two Ca^2+^ transport systems in isolated SVs; one is mediated by PMCAs at neutral pH, and the other is mediated by an independent Ca^2+^/H^+^ exchanger at alkaline pH. Our results strongly support a united model indicating that PMCAs are solely responsible for both activities in the majority of the SV population at neutral pH. Novel findings extending beyond those of previous studies^21–23,29^ are as follows. First, even at neutral pH of the extra-vesicular space, Ca^2+^ alkalized pre-acidified SV lumen. This phenomenon became evident when BAPTA was used to control free Ca^2+^ concentrations, although we currently do not understand why the use of EGTA attenuated alkalization signals in the acidification assays. Since the SV alkalization by Ca^2+^ was restored by vanadate which concomitantly impeded ^45^Ca uptake into SVs, we conclude that PMCAs are responsible for Ca^2+^/H^+^ exchange activity. This is consistent with more recent findings that counter transport of H^+^ is associated with Ca^2+^ transport by PMCAs^43^. Second, the Ca^2+^/H^+^ exchange at neutral pH exhibited high affinity for Ca^2+^ (*K*_m_ ~ 400 nM) that was similar to the reported values for PMCAs^44^, whereas previously reported Ca^2+^/H^+^ exchange activity at alkaline pH was characterized as a low affinity Ca^2+^/H^+^ exchanger (*K*_0.5_ ~ 217 µM)^22^. Third, although PMCAs have been characterized as residing in the plasma membrane, our imaging analysis revealed that PMCAs were potentially capable of recycling in an activity-dependent manner at presynaptic terminals. This observation was further substantiated by the fact that recycling of PMCA1-SEP depended exclusively on the presence of synaptobrevin/VAMP2 on the same vesicles, which is essential for stimulus-dependent exocytosis of SVs^45^.

Our results are highly consistent with biochemical evidence that, in addition to V-type H^+^ ATPases, vanadate-sensitive P-type ATPases constitute the major ATPases in cholinergic SVs from Torpedo electric organs^46^ as well as clathrin-coated vesicles from bovine brains^47^. Although PMCAs, which are P-type ATPases, have been characterized as plasma membrane residents, our results demonstrate that PMCAs constitute the major ATPase on recycling SVs as well. This is also consistent with proteomic analyses that identified PMCAs within SV fractions^34,48^. Thus, besides the main role of PMCAs in excluding Ca^2+^ from the presynaptic cytosol to the extracellular space at the plasma membrane, vesicular PMCAs may also contribute to Ca^2+^ clearance from the presynaptic cytosol. Furthermore, since Ca^2+^ transport into SVs is coupled to H^+^ efflux, vesicular PMCAs may be involved in the regulation of ΔµH^+^ of SVs, which drives neurotransmitter uptake into SVs. Indeed, external (cytosolic) Ca^2+^ strongly inhibited dopamine uptake into isolated SV fraction^49^, which is reasonable given that ΔpH, the driving force for dopamine uptake, is attenuated by Ca^2+^/H^+^ exchange. In contrast, glutamate uptake, which is predominantly driven by Δ**Ψ**, was only marginally facilitated under the condition where Δ**Ψ** predominated in the presence of 17 mM Cl^−^^49^. This subtle effect by Ca^2+^ on glutamate transport indicates that PMCAs on SVs are electro-neutral pumps (Ca^2+^:H^+^  = 1:2), which is consistent with observations in snail neurons^43^. Alternatively, the fact that glutamate transport in the presence of Ca^2+^ retained a typical biphasic Cl^−^ dependency^49^ (note that increasing Cl^−^ concentrations build up greater ΔpH) may indicate an important contribution of protons (or vesicular pH) on VGLUT function^2–4^. Further investigations will be needed to clarify how Ca^2+^/H^+^ exchangers in conjunction with vesicular Cl^−^ carriers, either VGLUTs or the ClC-type Cl^−^ transporters, affect glutamate transport into SVs.

The plasma membrane PMCAs are responsible for transient acidification of the cytoplasm during sustained stimulation at mouse motor nerve terminals, which may regulate endocytosis^31^. Our imaging analysis indicated that a substantial portion of vesicular PMCAs are inserted into the plasma membrane by exocytosis and retained there until endocytosis is completed, indicating that the total PMCA expression on the plasma membrane increases, especially during high activity. Although the contribution of the vesicular PMCAs that are inserted into the plasma membrane to acidification of the cytoplasm seems to be negligible^31^, it remains to be determined to what extent Ca^2+^ extrusion from the cytoplasm is accelerated by the vesicular PMCAs that translocate into the plasma membrane during high neural activity.

In mammals, four different genes encode the four isoforms of PMCA^50^. PMCA1 and PMCA4 are expressed ubiquitously, whereas PMCA2 and PMCA3 are expressed predominantly in the central nervous system. Furthermore, each isoform has multiple splice variants, comprising more than 30 spliced isoforms, although little is known about the functional significance of multiple splice variants^50^. In this study, we show that one of the PMCAs, the full length PMCA1 (1,249 a.a.) fused to pHluorin at its luminal region, is sorted preferentially to recycling SVs rather than to the plasma membrane at presynaptic terminals, and its distribution and recycling behaviors are very similar to those of the SV marker, synaptophysin (Figs 7 and 8). It remains unknown whether other PMCA isoforms or respective splice variants show similar properties to those of the full length PMCA1-pHluorin at presynaptic terminals, which may necessitate more complex regulation of Ca^2+^ and H^+^ dynamics at presynaptic terminals. Additionally, it should be kept in mind that we still lack concrete evidence for the existence of PMCA proteins on SVs, as the evidence we provide in this study relies on the usage of pharmacological blockade of Ca^2+^ transport by PMCA blockers and also on the exogenous expression of PMCA1 fused to fluorescent proteins. Although the SV proteome supports the existence of PMCA in the SV fraction isolated from native brains^32^, previous fractionation experiments, combined with western blot analysis using isoform-specific PMCA antibodies, have provided controversial results concerning the localization of PMCA isoforms in synaptic vesicles^49,50^, probably due to their predominant expression at the plasma membrane of the cell body. Thus, direct demonstration of PMCA isoforms on synaptic vesicles, e.g. via immuno-gold labeling of isolated SV fractions or immuno-gold labeling of brain sections, will be essential to confirm their vesicular localization in the future.

SV2s are postulated to function as a Ca^2+^ transporter, since synaptic phenotypes observed in SV2-deficient mice could be well explained if SV2s function as Ca^2+^ transporters^26,27^. However, our results from direct tests of Ca^2+^ transport into SV2-deficient vesicles rule out this hypothesis (Figs 4 and 6). Therefore, the Ca^2+^-related phenotypes observed previously in SV2-deficient mice may be indirect consequences of unknown functions of SV2. Of note, it has recently been shown that SV2A mediates galactose/H^+^ symport when heterologously expressed in yeast cells^51^. Although the functional significance of galactose in the nervous system, particularly its role in SVs, has been enigmatic, changes in either metabolism of carbohydrates or composition of glycans attached to proteins or lipids may indirectly regulate Ca^2+^ homeostasis at presynaptic terminals, which would lead to the observed Ca^2+^-related phenotypes in SV2-deficient synapses. Uncovering the link between SV2s’ function and presynaptic [Ca^2+^] regulation will help to elucidate the role of SV2s in epileptogenesis caused by SV2A gene knockout^26,52^.

In summary, our present results collectively suggest that Ca^2+^ transport across SV membranes is predominantly, if not exclusively, mediated by PMCAs. Due to their property as Ca^2+^/H^+^ exchangers, PMCAs may contribute to the regulation of ΔµH^+^ and the dynamic control of cytosolic Ca^2+^ and H^+^ at presynaptic terminals. Since various single nucleotide polymorphisms (SNPs) have been identified in PMCA genes that are associated with neuronal disorders such as autism and deafness^53^, it will be crucial to establish how these SNPs affect the function and distribution of PMCAs in neurons to elucidate the mechanisms underlying these diseases.

## Materials and Methods

### Synaptic vesicle isolation

Crude synaptic vesicle fraction (LP2) was isolated from mouse whole brains with slight modifications^54^. Briefly, whole brains from C57BL/6 mice were homogenized in homogenization buffer (320 mM sucrose, 4 mM MgSO_4_, 4 mM HEPES-NaOH, pH 7.3). The homogenate was centrifuged fro 10 min at 1,000 × *g*, the resulting supernatant (S1) was centrifuged for 15 min at 12,000 × *g*. The resulting pellet (P2) was washed with homogenization buffer and re-centrifuged for 15 min at 13,000 × *g* to obtain crude synaptosomes (P2′). To release SVs from the synaptosomes, P2′ fraction was subjected to an osmotic shock by the addition of 9 volume of ice-cold water and the subsequent homogenization. The resulting suspension was centrifuged for 20 min at 33,000 × *g*. After centrifugation, the supernatant (LS1; lysate supernatant) was centrifuged for 2 hours at 260,000 × *g*. The final membrane pellet (LP2) was resuspended in standard acidification buffer (300 mM sucrose, 4 mM MgSO_4_, 10 or 20 mM MOPS-KOH, pH 7.2), and stored at −80 °C until use. Essentially, all fluorometric assays, unless indicated otherwise, were performed with this fraction. For determination of apparent affinity of Ca^2+^/H^+^ exchange activity and radioactive^45^ Ca^2+^ uptake, LP2 obtained from Wistar rat brains was further purified by sucrose gradient centrifugation (50–800 mM continuous gradient). After centrifugation at 65,000 × *g*_*max*_ for 4 hours, turbid materials visible in the middle of the gradient (in the range of 200 to 400 mM sucrose) were pooled and sedimented by centrifugation at 260,000 × *g*_*max*_ for 90 min. The resulting pellet (SV) was resuspended in acidification buffer and stored at −80 °C until use.

### Calculation of free Ca^2+^ concentrations

Free calcium concentration was calculated by solving simultaneous equations in four unknowns: concentration of Ca^2+^ binding with BAPTA ([CaBAPTA]), concentration of Mg^2+^ binding with BAPTA ([MgBAPTA]), concentration of Mg^2+^ binding with ATP ([MgATP]) and that of Ca^2+^ binding with ATP ([CaATP]) as follows.

$${{\rm{K}}{\rm{^{\prime} }}}_{{\rm{C}}{\rm{a}}{\rm{B}}{\rm{A}}{\rm{P}}{\rm{T}}{\rm{A}}}={\textstyle \tfrac{[{\rm{C}}{\rm{a}}{\rm{B}}{\rm{A}}{\rm{P}}{\rm{T}}{\rm{A}}]}{({[{{\rm{C}}{\rm{a}}}^{2+}]}_{{\rm{T}}}-[{\rm{C}}{\rm{a}}{\rm{B}}{\rm{A}}{\rm{P}}{\rm{T}}{\rm{A}}]-[{\rm{C}}{\rm{a}}{\rm{A}}{\rm{T}}{\rm{P}}]{)([{\rm{B}}{\rm{A}}{\rm{P}}{\rm{T}}{\rm{A}}]}_{{\rm{T}}}-[{\rm{C}}{\rm{a}}{\rm{B}}{\rm{A}}{\rm{P}}{\rm{T}}{\rm{A}}]-[{\rm{M}}{\rm{g}}{\rm{B}}{\rm{A}}{\rm{P}}{\rm{T}}{\rm{A}}])}}$$

$${{\rm{K}}^{\prime} }_{{\rm{MgBAPTA}}}=\tfrac{[\mathrm{MgBAPTA}]}{({[{{\rm{Mg}}}^{2+}]}_{{\rm{T}}}-[{\rm{MgBAPTA}}]-[{\rm{MgATP}}]{)([\mathrm{BAPTA}]}_{{\rm{T}}}-[{\rm{CaBAPTA}}]-[{\rm{MgBAPTA}}])}$$

$${{\rm{K}}^{\prime} }_{{\rm{CaATP}}}=\tfrac{[\mathrm{CaATP}]}{({[{{\rm{Ca}}}^{2+}]}_{{\rm{T}}}-[{\rm{CaBAPTA}}]-[{\rm{CaATP}}]{)([\mathrm{ATP}]}_{{\rm{T}}}-[{\rm{CaATP}}]-[{\rm{MgATP}}])}$$

$${{\rm{K}}^{\prime} }_{{\rm{MgATP}}}=\tfrac{[\mathrm{MgATP}]}{({[{{\rm{Mg}}}^{2+}]}_{{\rm{T}}}-[{\rm{MgBAPTA}}]-[{\rm{MgATP}}])\,{([\mathrm{ATP}]}_{{\rm{T}}}-[{\rm{CaATP}}]-[{\rm{MgATP}}])}$$

[Ca^2+^]_T_, [Mg^2+^]_T_, [BAPTA]_T_ and [ATP]_T_ are the total concentrations of each substance. [Ca^2+^]_T_ is calculated from an equation ${[{{\rm{Ca}}}^{2+}]}_{{\rm{T}}}={[{{\rm{Ca}}}^{2+}]}_{{\rm{add}}}+{[{{\rm{Ca}}}^{2+}]}_{{\rm{ctm}}}$, where [Ca^2+^]_add_ and [Ca^2+^]_ctm_ are concentration of added CaCl_2_ and of contaminated Ca^2+^ determined routinely by fura-2 assay, respectively. *K′*_CaBAPTA_, *K′*_MgBAPTA_, *K′*_CaATP_ and *K′*_MgATP_ are the overall apparent association constants. Microsoft Excel Solver was used to solve the equations.

To set up the equations above, the overall apparent association constants, *K′*s (*K′*_CaBAPTA_, *K′*_MgBAPTA_, *K′*_MgATP_ and *K′*_CaATP_) were converted from the absolute association constants, *K*s, which were determined for standard conditions (see details in Marks and Maxfield^55^). Supplementary Table 1 lists the published association constants for BAPTA and ATP, as well as the *ΔH* values for BAPTA and ATP. *ΔH* values were necessary for the conversions from the absolute association constants to the overall apparent association constants.

### Acidification assay

Acidification measurements were performed according to previous publications using acridine orange (AO, Molecular Probes) as a ΔpH reporter^3^. Changes in AO fluorescence (excitation at 492 nm and emission at 530 nm with slit lenghs with 2.5 nm, HMT 700 V) were monitored in a Hitachi F2500 fluorometer (Hitachi, Japan) at 32 °C, unless otherwise stated^3^. Typically, 20 µg of LP2 or SV fraction was preincubated in 1 mL of assay buffer (300 mM sucrose, 4 mM MgSO_4_, 1.5 µM AO, 10 or 20 mM MOPS, pH 7.2) with varying composition of 5 mM K-glutamate, 3 mM or 100 mM KCl, 50 µM EGTA, and 50 µM BAPTA as indicated in the figures or figure legends. After a stable baseline was achieved (usually within 10 min), 2 mM ATP was added to start acidification. Various concentrations of CaCl_2_ or 50 µM other divalent cations were added at 10 min where indicated. At the end of recordings, a V-ATPase inhibitor, bafilomycin A_1_ (500 nM) was added to ensure that quenching of AO was due to proton translocation by the V-ATPase. For Figs 4 and 5, 15 µM cyclopiazonic acid, 500 µM vanadate, or 30 µM levetiracetam was pre-incubated for 5 min before measurements. Representative traces from multiple measurements are shown in the figures. For estimation of temperature co-efficient (Q_10_) for the Ca^2+^-dependent AO de-quenching, acidification assays were performed at two different temperatures. The Q_10_ was calculated from an equation:

$${{\rm{Q}}}_{10}=(\frac{{\tau }_{1}}{{\tau }_{2}}{)}^{(\frac{10}{{{\rm{T}}}_{2}-{{\rm{T}}}_{1}})},$$

in which τ_1_ and τ_2_ are time constants of recovery phases of acridine orange fluorescence after the addition of Ca^2+^ at temperature T_1_ and T_2_. τ_1_ and τ_2_ were obtained by a first order exponential fitting using a Solver function in Excel software. T_1_ and T_2_ (T_2_ > T_1_) are 32.3 and 37.3 °C, respectively.

### Fura-2 assay

Ca^2+^ concentrations in some experimental solution were measured using fura-2 (pentapotassium salt, impermeant, Invitrogen) as a Ca^2+^ reporter. Changes in fura-2 fluorescence (excitation at 340 nm and emission at 510 nm, with slit lenghs with 2.5 nm, HMT 400 V) were monitored in a Hitachi F2500 fluorometer (Hitachi, Japan) at 32 °C. 1 ml of an assay buffer (100 mM KCl, 10 mM MOPS-KOH, pH7.2) was preincubated for 10 min. The preincubation was followed by adding 300, 600, 900, 1200 and 1500 nM total exogenous CaCl_2_ each with 30 sec intervals. At the end of recordings, 50 μM BAPTA was added to chelate exogenous and contaminated Ca^2+^.

### Animals

C57BL/6NJcl mice were purchased from CLEA, Japan. ICR mice were purchased from SLC, Japan. All mice were given food and water *ad libitum*. Animals were kept in an SPF facility with a 12-hour light and 12-hour dark cycle. The ambient temperature was maintained around 21 °C with a relative humidity of 50%. ICR mice (12 to 20 weeks old) were used as recipients. A combination anesthetic (0.75 mg/kg of medetomidine, 4.0 mg/kg of midazolam, and 5.0 mg/kg of butorphanol) was used for surgery. The anesthetics were administered to recipient mice by intraperitoneal injection. All animal experiments were approved by the Institutional Animal Care and Use Committee of the RIKEN Kobe branch (approval number: QA2013-04-6) and the Institutional Animal Care and Use Committee of Doshisha University.

### One-cell embryo microinjection

C57BL/6N females (4–6 weeks old) were superovulated and mated with C57BL/6N males. Fertilized eggs were collected from the ampulla of the oviduct of plugged C57BL/6N females by micro-dissection and kept in KSOM medium (Merck Millipore) in a 5% CO_2_ incubator at 37 °C. *Cas9* mRNA (100 ng/µL) and six gRNAs (50 ng/µL each, 300 ng/µL total) were co-injected into the cytoplasm of fertilized eggs in M2 medium (Merck Millipore) at room temperature. Details of the cytoplasmic injection procedure have been described previously^56^. After microinjection, the injected embryos were cultured for 1 hr in KSOM medium (Merck Millipore) in a 5% CO_2_ incubator at 37 °C, then 15–30 embryos were transferred to the oviducts of recipient ICR female mice.

### One-step generation of double gene knockouts of SV2A/2B and SV2B/2C

Double gene knockout (DKO) mice of SV2A/2B and SV2B/2C were generated by the triple-target CRISPR method^57^. Briefly, *Cas9* mRNA and sgRNAs were synthesized according to the protocol reported previously^57^. All gRNAs were selected from pre-made design in Database (http://crispr.riken.jp). *Cas9* mRNA (100 ng/µL) and six gRNAs (50 ng/µL each, 300 ng/µL total) were injected into the cytoplasm of fertilized eggs of C57BL/6NJcl mice. For SV2A/2B DKO, six gRNA targets were used (Sv2a_8 5′-AAGGCGAACGCATGGCAGAT-3′, Sv2a_9 5′-GCGTAAAGATCGGGAAGAAT-3′, Sv2a_25 5′-GGCAGCCTTCCTTATTGTGC-3′, Sv2b_28 5′-CTGGCAATCGAAGGGCAATC-3′, Sv2b_38 5′-GTGGACCCTCTTCTTCGTCT-3′, Sv2b_41 5′-AGGTATCGGGACAACTATGA-3′). For SV2B/2C DKO, six gRNA targets were used (Sv2b_28, Sv2b_38, Sv2b_41, Sv2c_56 5′-ACTGGAATGGAATACGAGAA-3′, Sv2c_77 5′-AGACCTATGCATACCAAATT-3′, Sv2c_78 5′-CACAAACACCTCCACGCCAT-3′).

### Ca^2+^ transport assay

The concentrations of SV or LP2 fractions were adjusted to 0.2 mg/mL in sucrose buffer. 100 µL aliquots were preincubated for 10 min at 32 °C. The reaction was started by addition of 10 µM (final concentration) ^45^Ca^2+^ dissolved in sucrose buffer containing ATP (2 mM), vanadate (500 µM), eosinY (10 µM), cyclopiazonic acid (15 µM) and levetiracetam (30 µM) when indicated. All incubations contained 0.125% (v/v) DMSO and 0.167% (v/v) ethanol in a final volume of 150 µL. After incubation for 10 min at 32 °C, the reaction was stopped by addition of 3 mL of ice-cold buffer, followed by filtration through nitrocellulose filters. The incubation tubes were washed out with 3 mL of ice-cold buffer, and the wash out buffer was poured through the filter. The filters were washed out twice with 2.5 mL of ice-cold buffer. Radioactivity retained on filters was measured by liquid scintillation counting using an ALOKA LSC-6100 liquid scintillation counter (ALOKA, Japan).

### Western blot analysis

SDS-PAGE was used to separate 20 µg of LP2 fractions from wild-type or SV2-DKO mice. Proteins were transferred to a PVDF membrane. The resulting blots were probed with isoform specific antibodies for SV2s (Synaptic Systems, Germany) or with a pan-SV2 monoclonal antibody (a kind gift from Reinhard Jahn, Göttingen, Germany). For detection, the appropriate secondary antibodies conjugated to horseradish peroxidase were used. After washing steps, the horseradish peroxidase was detected by enhanced chemiluminescence using a commercially available kit (Perkin Elmer, Inc., MA). As loading controls, anti-synaptophysin monoclonal antibody (Cl7.2, ‘a gift from Reinhard Jahn’ instead of ‘Synaptic Systems, Germany’) was used.

### Molecular biology

To construct PMCA1-SEP, a full length mouse PMCA1 (accession no. NM_001359506.1) was amplified by PCR from mouse adult brain cDNA generated using SuperScript RT-PCR system (Invitrogen) and subcloned into a *Stu*I site of pCR-Blunt vector (Thermo Fisher Scientific). A DNA fragment encoding the N-terminal region of PMCA1 (a.a. 1–139) with linker sequence (STSGGSGGTGGS) and a fragment of super-ecliptic pHluorin (SEP) amplified from SypHy plasmid^58,59^ (a kind gift from Leon Lagnado) were amplified by PCR and cloned into an *Eco*RV site of pcDNA3.1(+) using In-Fusion Cloning Kit (Clontech). A DNA fragment encoding the C-terminal region of PMCA1 (a.a. 140-1,220) was PCR-amplified and cloned into a *Not*I site of pcDNA3.1(+) which contained the N-terminal region of PMCA1 and SEP using In-Fusion Cloning Kit. To construct Syntaxin1a-SEP, a full length mouse Syntaxin-1a (accession no. NM_016801.3) without the stop codon and a fragment of SEP were amplified by PCR and simultaneously cloned into a *Nhe*I/*Eco*RV site of pcDNA3.1(+). A DNA fragment encoding SypHy^59^ was amplified by PCR and subcloned into a *Nhe*I/*Xba*I site of pcDNA3.1(+).

### Neuronal cultures

Primary hippocampal cultures were prepared from embryonic day 16 ICR mice as described previously^60^ with slight modifications. Briefly, hippocampi were dissected and were incubated with papain (90 units/mL, Worthington) for 20 min at 37 °C. After digestion, hippocampal cells were plated onto poly-D-lysine-coated coverslips in 24- or 12-well plates (Falcon) at a density of 20,000 cells/cm^2^ and kept in a 5% CO_2_ humidified incubator. At 2–4 days *in vitro* (DIV), 40 μM FUDR (Sigma) and 100 μM uridine (Sigma) were added to inhibit the growth of glial cells. One-fifth of the culture medium was replaced with fresh medium every 2–4 days. Cultures were transfected with plasmids encoding either PMCA1-SEP, SypHy, or Syntaxin-1a-SEP at 5–7 DIV using CalPhos^TM^ mammalian transfection kit (Clontech) in accordance with a calcium phosphate transfection method which is optimized for neuronal cultures^61^, and were subjected to experiments at 12–14 DIV. Animals for the primary neuron cultures were treated according to our institutional guidelines for the care and use of animals (Doshisha University).

### Immunostaining

At DIV12, neural cells were fixed with 4% (wt/vol) paraformaldehyde in phosphate buffer (Wako) for 10 min at room temperature (RT). After washing with phosphate buffered saline (PBS), the neurons were permeabilized with PBS containing 0.1% Triton X-100 for 20 min at RT, and incubated with PBS containing 10% (vol/vol) fetal bovine serum (FBS) and 0.1% Triton X-100 for 30 min at RT. The cells were incubated with rabbit polyclonal anti-GFP antiserum (1:1,000) and mouse monoclonal anti-synaptophysin antibody (1:1,000; C17.2) (both were kind gifts from Reinhard Jahn) for 1 hour at RT. The cells were rinsed three times with PBS, and further incubated with Alexa-488-conjugated anti-rabbit IgG (1:1,500; Invitrogen) and Alexa-555-conjugated anti-mouse IgG (1:1,500; Invitrogen) for 30 min at RT. After washing steps, Alexa-488 or Alexa-555 fluorescence was acquired with 470/22 nm excitation and 514/30 nm emission filters or 556-to 570-nm excitation and 600- to 650-nm emission filters, respectively.

### Live imaging

Cells cultured on a glass coverslip were placed in a custom-made imaging chamber on a movable stage and continuously perfused with standard extracellular solution containing (in mM): 140 NaCl, 2.4 KCl, 10 HEPES, 10 glucose, 2 CaCl_2_, 1 MgCl_2_, 0.02 CNQX, and 0.025 D-APV (pH 7.4). A solution containing 50 mM NH_4_Cl (pH 7.4) was applied directly onto the area of interest with a combination of a fast flow exchange microperfusion device and a bulb controller, both of which were controlled by Clampex 10.2. To estimate luminal pH and surface fraction of the SEP probes, a MES-buffered solution at pH 5.5 and 50 mM NH_4_Cl (pH 7.4) were successively applied to cultured neurons as described previously^35^. Briefly, SEP fluorescence during acid quenching (F_Q_) and during subsequent NH_4_Cl application (F_NH4Cl_) was measured. The observed fluorescence in a given terminals is thought to be the sum of the fluorescence derived from the surface fraction of the probes (S) that experiences extracellular pH and from the vesicular fraction (1 − S) that is exposed to luminal pH (pHv). The fluorescence during the baseline (F_0_) and the fluorescence during F_Q_ are expressed as

1 $${{\rm{F}}}_{0}={\rm{S}}\times {{\rm{F}}}_{{\rm{pH}}7.4}+(1-{\rm{S}})\times {{\rm{F}}}_{{\rm{pHv}}}$$

2 $${{\rm{F}}}_{{\rm{Q}}}={\rm{S}}\times {{\rm{F}}}_{{\rm{pH}}5.5}+(1-{\rm{S}})\times {{\rm{F}}}_{{\rm{pHv}}}$$

where F_pH 5.5_ and F_pHv_ are the total fluorescence values predicted when all probe molecules in a terminal are exposed to pH 5.5 and pH_v_, respectively. By solving Equations (1) and (2), S and F_pHv_/F_pH 7.4_ were calculated as follows:

$${\rm{S}}=({{\rm{F}}}_{0}/{{\rm{F}}}_{{\rm{pH}}7.4}-{{\rm{F}}}_{{\rm{Q}}}/{{\rm{F}}}_{{\rm{pH}}7.4})/(1-{{\rm{F}}}_{{\rm{pH}}5.5}/{{\rm{F}}}_{{\rm{pH}}7.4}),$$

$${{\rm{F}}}_{{\rm{p}}{\rm{H}}{\rm{v}}}{/{\rm{F}}}_{{\rm{p}}{\rm{H}}7.4}=({{\rm{F}}}_{0}/{{\rm{F}}}_{{\rm{p}}{\rm{H}}7.4}-{\rm{S}})/(1-{\rm{S}}).$$

According to the Henderson-Hasselbalch equation, F_pH 5.5_/F_pH 7.4_ was given as follows:

$${{\rm{F}}}_{{\rm{p}}{\rm{H}}5.5}{/{\rm{F}}}_{{\rm{p}}{\rm{H}}7.4}=(1/(1+{10}^{{\rm{p}}Ka-5.5}))/(1/(1+{10}^{{\rm{p}}Ka-7.4})),$$

where the p*K*_a_ value of SEP estimated previously by using SypHy was 7.1^53^.

Finally, SV pH (pH_v_) was then calculated as follows:

$${{\rm{p}}{\rm{H}}}_{{\rm{v}}}={\rm{p}}{K}_{{\rm{a}}}-\,{\rm{l}}{\rm{o}}{\rm{g}}((1+{10}^{{\rm{p}}Ka-7.4})/{{\rm{F}}}_{{\rm{p}}{\rm{H}}{\rm{v}}}/{{\rm{F}}}_{{\rm{p}}{\rm{H}}7.4}-1).$$

Fluorescence imaging was carried out at room temperature (~27 °C) on an inverted microscope (Olympus) equipped with a 60× (1.35 NA) oil immersion objective and 75 W Xenon lamp. Images (1024 × 1024 pixels) were acquired with a scientific cMOS camera (ORCA-Flash 4.0, Hamamatsu Photonics) with 100 ms exposure time under the control of MetaMorph software (Molecular Devices). Fluorescence of SypHy, PMCA1-SEP or Syntaxin-1a-SEP was imaged with 470/22 nm excitation and 514/30 nm emission filters.

For live imaging, images were acquired in time-lapse mode at 1 Hz under control of MetaMorph software. Field stimulation was delivered via bipolar platinum electrodes with 1 msec constant voltage pulses (50 V). A solution containing 50 mM NH_4_Cl was applied at the end of recordings as described above.

### Study approval

All experiments in this study were carried out under the approval of Doshisha University Animal Committee and Recombinant DNA Experiments Committee, and of the Institutional Animal Care and Use Committee of the RIKEN Kobe branch (approval number: QA2013-04-6). All experiments were performed in accordance with the guidelines and regulations of the respective institutions.

## Supplementary information

Supplementary materials

## Data Availability

The data that support the findings of this study are available from the corresponding authors upon reasonable request.
