# Supplementary material for: Expression of plasma membrane calcium ATPases confers Ca2+/H+ exchange in rodent synaptic vesicles
Source: Sci Rep. 2019 Mar 12;9:4289. doi: 10.1038/s41598-019-40557-y (PMC6414521; doi:10.1038/s41598-019-40557-y)
Supplement: Supplementary file 1 — Supplementary materials [file 41598_2019_40557_MOESM1_ESM.pdf]

Supplementary materials

Expression of plasma membrane calcium ATPases confers Ca^{2+}/H^{+} exchange in rodent synaptic vesicles

Yoshiyasu Ono, Yasunori Mori, Yoshihiro Egashira, Kenta Sumiyama and Shigeo Takamori

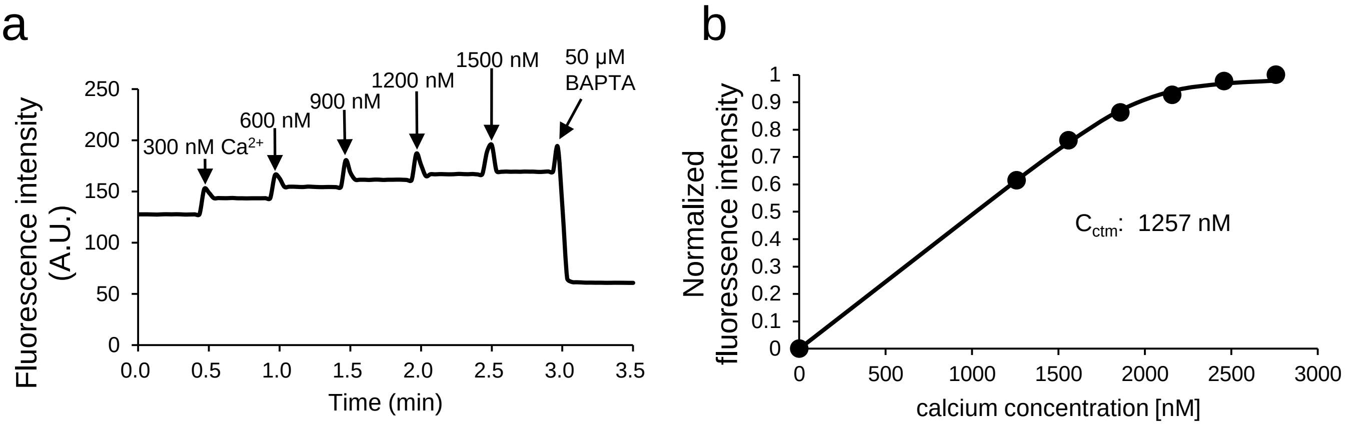

Supplementary figure 1.
Contaminated Ca^{2+} in experimental solutions was determined using a fura-2 assay.
(a) Fluorescence intensity of fura-2 in a cuvette with various concentrations of Ca^{2+}. 1 ml of assay buffer (100 mM KCl, 10 mM MOPS-KOH, pH7.2) was preincubated for 10 min. The preincubation was followed by adding 300, 600, 900, 1200 and 1500 nM of total exogenous $CaCl_2$. Finally, 50 μM BAPTA was added at the time points indicated by the arrows.
(b) Fluorescence intensity (FI) above the intensity of fluorescence with 50 μM BAPTA was plotted against the added Ca^{2+} concentrations. FI was normalized by maximum FI obtained at 1500 nM exogenous Ca^{2+}. The data points were fitted with the following equation (see detail in Iino [1]):

$$FI = \left[C_T + C_f + K_d - \left\{\left(C_T + C_f + K_d\right)^2 - 4C_T C_f\right\}^{1/2}\right] / \left(2C_f\right),$$
$$\text{where } C_T = C_{add} + C_{ctm}$$

C_T, C_{add}, C_{ctm}, C_f and K_d are concentrations of total Ca^{2+} including contaminated Ca^{2+}, of exogenously added Ca^{2+}, of contaminated Ca^{2+} in assay buffer, of added fura-2, and the dissociation constant of Ca·fura-2, respectively. Microsoft Excel Solver was used to solve the equations.
As a result, the C_{ctm} was 1257 nM.

In addition, the C_{ctm} of the assay buffer without KCl was estimated in the same procedure. The C_{ctm} in this situation was 875±9.32 (s.e.m., n = 3) nM. Moreover, the C_{ctm} of ultra pure water (MilliQ) was estimated. The C_{ctm} in this situation was 740±41.3 (s.e.m., n = 4) nM. Taking the above results of the C_{ctm} into consideration, we regarded C_{ctm} as 1 μM in the calculation of free Ca^{2+} concentration in Fig.2.

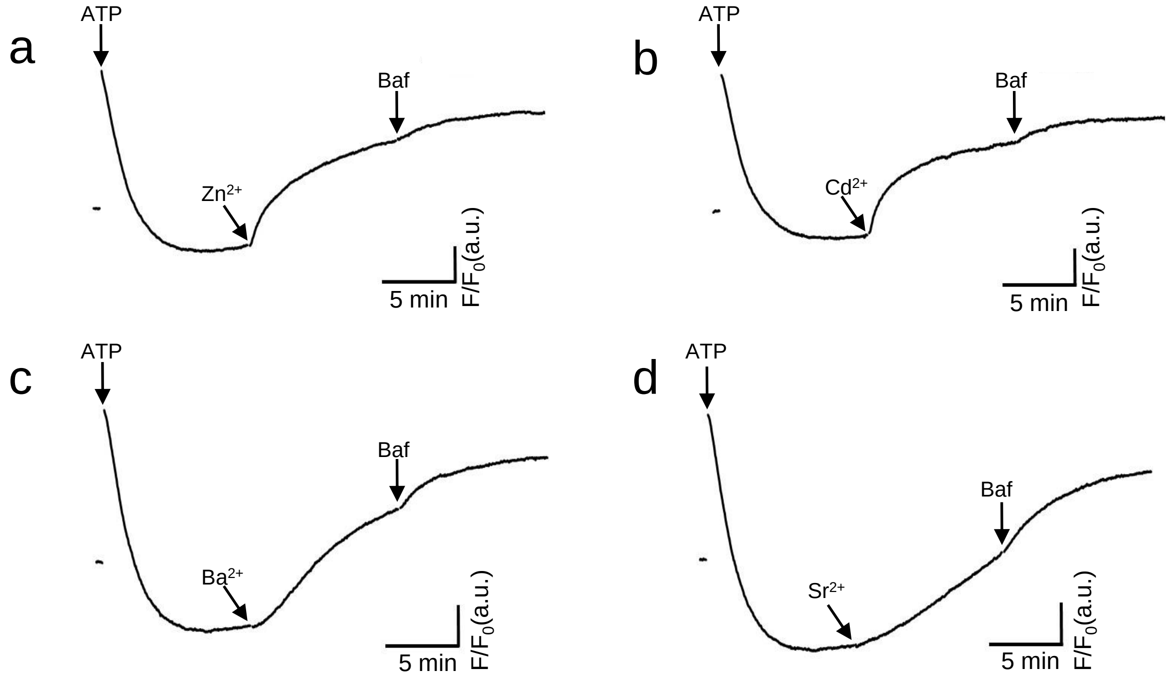

Supplementary figure 2.
LP2 fraction was acidified in the presence of 5 mM glutamate and 3 mM KCl at pH 7.2. After stable baselines were achieved, various divalent cations including Zn^{2+}, Cd^{2+}, Ba^{2+}, and Sr^{2+} at final concentration of 50 µM were added.

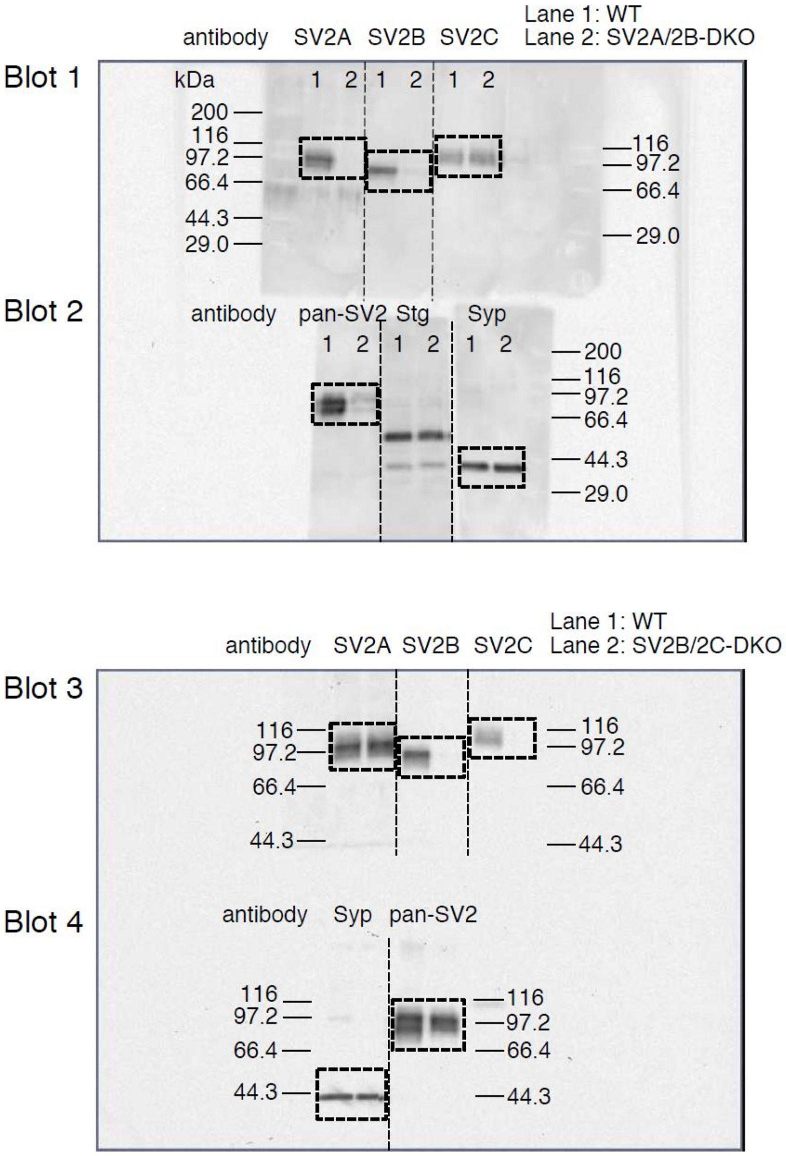

Supplementary figure 3.
Upper panel shows two original blots to produce the left panel of Fig. 4a. The blots were cut into pieces that contained protein samples from wild-type and SV2A/2B-DKO brains. The blot with antibody against Synaptotagmin 1 (Stg) was not used for the main figure. The areas used for the main figures are indicated by botted squares. Molecular weight standards (Protein Molecular Weight Marker (Broad), Takara Bio Inc., Japan) are indicated at the side of the blots. Bottom pannel shows two original blots to produce the right panel of Fig. 4a. The blots were cut into pieces that contained protein samples from wild-type and SV2B/2C-DKO mice.
Antibodies used were indicated at the top of the blots. Rabbit polyclonal antibodies against SV2A, SV2B, and SV2C were purchased from Synaptic Systems (see https://www.sysy.com/ and references therein). A mouse monoclonal antibody against synaptophysin (Syp) [2] and a monoclonal antibody that recognizes all SV2 isoforms (pan-SV2) [3] were kind gifts from Reinhard Jahn (Göttingen, Germany).

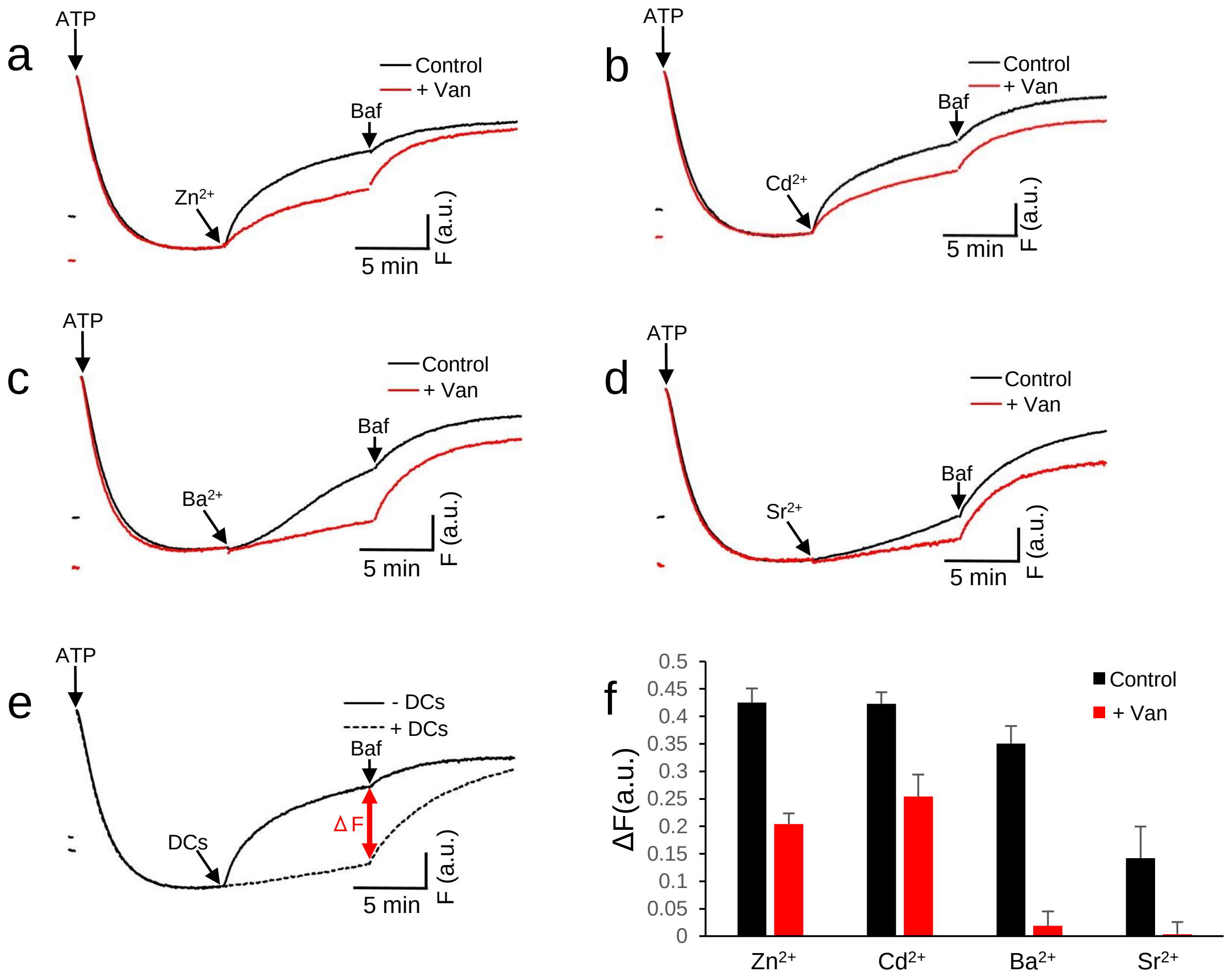

Supplementary figure 4.

Effects of vanadate (Van, 500 µM) on divalent cations (DCs)-induced alkalization. Vesicles were pre-acidified in the presence of 5 mM glutamate and 3 mM KCl in the absence (black) and presence (red) of 500 µM Van, and 50 µM (a) $ZnCl_2$, (b) $CdCl_2$, (c) $BaCl_2$ or (d) $SrCl_2$ were added to induce alkalization. Since vanadate slightly reduced AO quenching signals in the presence of 5 mM glutamate and 3 mM KCl, fluorescence traces were normalized by amplitudes of acidification. All traces are the representative data from three measurements in each condition. To quantitate effects of the vanadate on DCs-induced alkalization, the magnitudes of the alkalization with or without the vanadate were estimated. An example of the magnitudes, ΔFs, was indicated in a graph (e). The amplitudes in each condition were summarized in (f). Error bars indicate s.e.m. of three independent measurements.

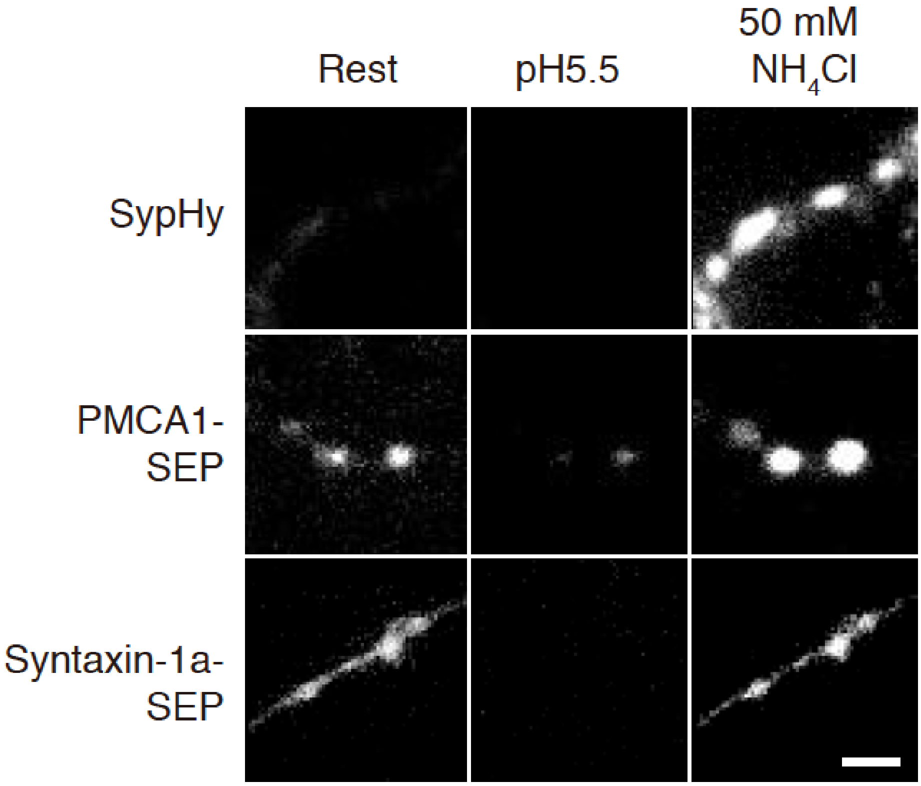

Supplementary figure 5.
Representative images of SypHy (upper panels), PMCA1-SEP (middle panels), and Syntaxin1a-SEP (lower panels) at resting condition (left), during acid (middle), and 50 mM NH_4Cl (right) application. Scale bar indicates 10 μm.

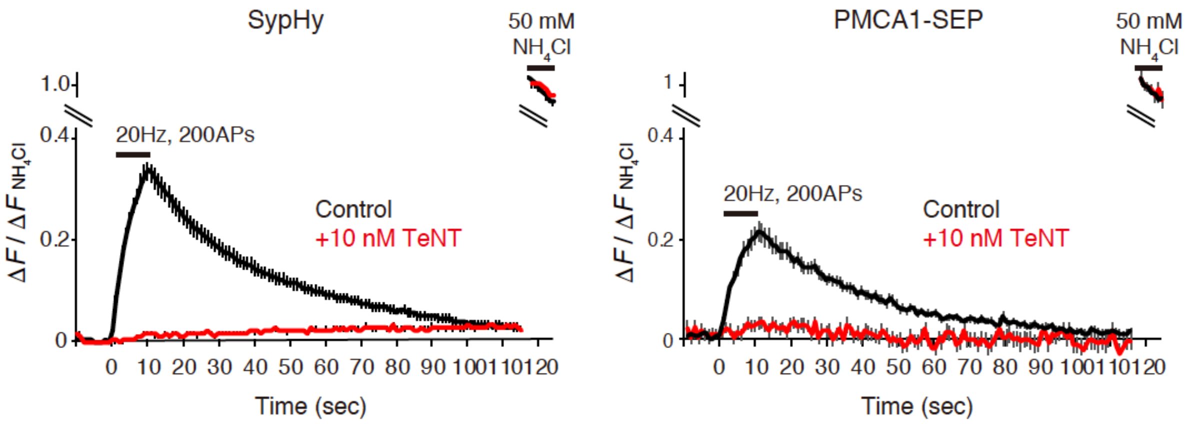

Supplementary figure 6.
Average traces of SypHy (left) and PMCA1-SEP (right) in control (black) and after treatment with 10 nM TeNT (red). Neurons were subjected to 20 Hz stimulation for 10 s. Fluorescence was normalized to that in the presence of 50 mM NH_4Cl at the end of the recordings.

Equilibrium	log $K^{(a)}$	$\Delta H^{(b)}$
$Ca^{2+} + BAPTA^{4-} \leftrightarrows CaBAPTA^{2-}$	6.97	13900 [J/mol]
$Mg^{2+} + BAPTA^{4-} \leftrightarrows MgBAPTA^{2-}$	1.77	53800 [J/mol]
$H^{+} + BAPTA^{4-} \leftrightarrows HBAPTA^{3-}$	6.36	
$H^{+} + HBAPTA^{3-} \leftrightarrows H_2BAPTA^{2-}$	5.47	
$Ca^{2+} + ATP^{4-} \leftrightarrows CaATP^{2-}$	3.828	2107 [cal/mol]
$Mg^{2+} + ATP^{4-} \leftrightarrows MgATP^{2-}$	2.144	4245 [cal/mol]
$H^{+} + ATP^{4-} \leftrightarrows HATP^{3-}$	6.476	
$H^{+} + ATP^{3-} \leftrightarrows H_2ATP^{3-}$	4.039	
$Ca^{2+} + HATP^{3-} \leftrightarrows HATP^{-}$	2.144	
$Mg^{2+} + HATP^{3-} \leftrightarrows HATP^{-}$	2.299	

Supplementary table 1

(a) Association constants for BAPTA (22 ºC, I = 0.1 M) are from Tsien[4]. Values for ATP (20 ºC, I = 0.1 M) are from MAXCHELATOR[5].

(b) A ΔH value for Ca^{2+} binding to BAPTA is from Harrison & Bers[6]. A value for Mg^{2+} binding to BAPTA is from Marks & Maxfield[7]. Values for either Ca^{2+} or Mg^{2+} binding to ATP are from Wilson & Chin[8].

References for Supplementary materials

1 Iino, M. Calcium-induced calcium release mechanism in guinea pig taenia caeci. *J. Gen. Physiol.* **94**, 363-383 (1989).

2 Jahn, R., Schiebler, W., Ouimet, C. & Greengard, P. A 38,000-dalton membrane protein (p38) present in synaptic vesicles. *Proc. Natl. Acad. Sci. USA* **82**, 4137-4141 (1985).

3 Buckley, K. & Kelly, R. B. Identification of a transmembrane glycoprotein specific for secretory vesicles of neural and endocrine cells. *J. Cell Biol.* **100**, 1284-1294 (1985).

4 Tsien, R. Y. New calcium indicators and buffers with high selectivity against magnesium and protons: design, synthesis, and properties of prototype structures. *Biochemistry* **19**, 2396-2404 (1980).

5 http://maxchelator.stanford.edu/xlsconstants.htm

6 Harrison, S. M. & Bers, D. M. The effect of temperature and ionic strength on the apparent Ca-affinity of EGTA and the analogous Ca-chelators BAPTA and dibromo-BAPTA. *Biochim. Biophys. Acta.* **925**, 133-143 (1987).

7 Marks, P. W. & Maxfield, F. R. Preparation of solutions with free calcium concentration in the nanomolar range using 1,2-bis(o-aminophenoxy)ethane-N,N,N',N'-tetraacetic acid. *Anal. Biochem.* **193**, 61-71 (1991).

8 Wilson, J. E. & Chin, A. Chelation of divalent cations by ATP, studied by titration calorimetry. *Anal. Biochem.* **193**, 16-19 (1991).
